# Hearing brain evaluated using near-infrared spectroscopy in congenital toxoplasmosis

**DOI:** 10.1038/s41598-021-89481-0

**Published:** 2021-05-12

**Authors:** Ana Lívia Libardi Bertachini, Gabriela Cintra Januario, Sergio Luiz Novi, Rickson Coelho Mesquita, Marco Aurélio Romano Silva, Gláucia Manzan Queiroz Andrade, Luciana Macedo de Resende, Débora Marques de Miranda

**Affiliations:** 1 Department of Pediatrics, Universidade Federal de Minas Gerais, Belo Horizonte, Brazil; 2 “Gleb Wataghin’’ Institute of Physics, University of Campinas, Campinas, Brazil; 3 Department of Mental Health, Universidade Federal de Minas Gerais, Belo Horizonte, Brazil; 4 Department of Speech and Hearing Therapy, Universidade Federal de Minas Gerais, Belo Horizonte, Brazil; 5 NUPAD – Center for Newborn Screening and Genetic Diagnostics, UFMG – Universidade Federal de Minas Gerais, Belo Horizonte, Brazil; 6 Centro de Tecnologia Em Medicina Molecular, Universidade Federal de Minas Gerais, Av. Prof. Alfredo Balena 190, Santa Efigênia, Belo Horizonte, MG 30130-100 Brazil

**Keywords:** Neurological disorders, Diseases of the nervous system, Sensory processing, Infectious diseases, Paediatric research

## Abstract

Congenital toxoplasmosis (CT) is a known cause of hearing loss directly caused by Toxoplasma gondii. Hearing loss might result from sensory, neural, or sensorineural lesions. Early treated infants rarely develop hearing loss, but retinochoroidal lesions, intracranial calcifications and hydrocephalus are common. In this study, we aimed to evaluate the brain evoked hemodynamic responses of CT and healthy infants during four auditory stimuli: mother infant directed speech, researcher infant directed speech, mother reading and researcher recorded. Children underwent Transitionally Evoked Otoacoustic Emission Auditory Testing and Automated Brainstem Auditory Response tests with normal auditory results, but with a tendency for greater latencies in the CT group compared to the control group. We assessed brain hemodynamics with functional near-infrared spectroscopy (fNIRS) measurements from 61 infants, and we present fNIRS results as frequency maps of activation and deactivation for each stimulus. By evaluating infants in the three first months of life, we observed an individual heterogeneous brain activation pattern in response to all auditory stimuli for both groups. Each channel was activated or deactivated in less than 30% of children for all stimuli. There is a need of prospective studies to evaluate if the neurologic or auditory changes course with compromise of children outcomes.

## Introduction

Congenital toxoplasmosis (CT) is an infectious parasitic disease caused by *Toxoplasma gondii*. The disease occurs when the infection is transmitted from the mother to the child during pregnancy. The incidence of CT varies with sociodemographic and cultural features. The reported incidence rates of CT are 6 to 8:10,000 births in North America and Europe; 20 to 24:10,000 in Africa; and 18 to 34:10,000 in South America^1^. In Brazil, CT’s frequency also varies nationwide, from 3 to 13:10,000 births^2–4^. The global estimated incidence of CT is 190,100 annual cases (15:10,000 births) with a global burden calculated as 1.20 million disability-adjusted life year (DALY)^1^.

Most children with CT are asymptomatic at birth, but some may have ophthalmic, neurological, or hearing injuries, configuring a broad spectrum of severity in clinical presentation^5,6^. Retinochoroiditis, cerebral calcification, and hydrocephalus might happen^6,7^. Early diagnosis and appropriate treatment reduce its severity, minimizing the occurrence of clinical manifestations^7–9^. In a cohort led by our group, 146,307 newborns were screened for CT over a seven-month period. *T gondii* infection was confirmed in 190 cases, and about 80% had retinochoroidal lesions, 20% intracranial calcifications, 6% hydrocephalus, 5% microcephaly^2^ and 4% sensorioneural hearing loss^10^.

The prevalence of sensorineural hearing loss (SNHL) in children who received appropriate treatment in the first year of life is rare, whereas it can reach 28% in children without effective treatment^11^. Autopsy of the temporal bones of subjects with CT revealed parasites in the internal auditory canal, the spiral ligament, stria vascularis and saccular macula suggesting that the hearing loss in subjects with CT is directly mediated by *T gondii* and can result in sensory, neural or sensorineural lesions^12^. CT was associated to worse performance on intellectual function, intelligence and adaptive function in comparison to health controls^13^. In animal models, mice with CT infection presented learning and memory impairment^14,15^. In the presence of hearing impairment, especially the retrocochlear, there is an increased risk for changes in auditory processing and language development, and performance. As a white matter disorder, CT might compromise development due to impairments in auditory and language processing.

In Resende et al., 106 children early treated for CT were evaluated. In this group, 27.4% presented central hearing dysfunction and 26.4% presented a language delay followed up 12 months of age^10^. Ferreira et al. observed higher latency in cortical responses to speech in CT children^16^. Children with CT have five times more risk to have an abnormality in brainstem auditory evoked potential and a higher latency of wave V^17^. It is worth noting that wave V latency reveals neurodevelopmental status of brainstem pathway, and it is an important marker of hearing sensitivity in the newborn^18^. Although the above-mentioned works advanced our current understanding on the CT effects to the brain, further details concerning their impact are still of debate. Better understanding of the main mechanisms related to the changes in the central auditory system and their associated deficits due to CT is critical to improve children outcomes.

In this work, we assessed the hemodynamics of the brain through functional near-infrared spectroscopy (fNIRS) measurements. By noninvasively shining near-infrared light in the scalp, fNIRS is able to recover oxy- (HbO) and deoxy-hemoglobin (HbR) concentration changes in the brain cortex. These changes are related to brain activity due to neurovascular coupling and allow quantitative and qualitative evaluation of the cortical activity^19–21^. Together, these features allowed us to measure the infant’s brain activity in a more natural environment (e.g., when they were not sedated)^21–23^. In the present study, we aimed to observe the brain evoked hemodynamic response to auditory stimuli in healthy and CT infants in the first months of life. Motivated by previous studies^22^, we hypothesized that children with CT have distinct patterns of brain activation compared to healthy children in response to auditory stimuli in the first months of life.

## Results

### Hearing assessment

All children included in the study presented normal hearing results. In Table 1, we can see that the average value of wave V of the AABR (Automated Brainstem Auditory Response) in both ears was higher in the CT group when compared to the control group.

**Table 1:** Descriptive analysis of the wave V findings of the AABR in the study groups.

	AABR (RE) (ms)		AABR (LE) (ms)	
	CT	C	CT	C
Valid	38	20	38	20
Mean	7.592	7.260	7.543	7.418
Std. Deviation	0.5430	0.4814	0.4290	0.5499
Minimum	6.710	6.120	6.400	6.180
Maximum	9.500	8.200	8.400	8.600

*AABR* automated brainstem auditory response; *RE* right ear; *LE* left ear; *ms* milliseconds; *CT* congenital toxoplasmosis group; *C* control group.

**Figure 1:**
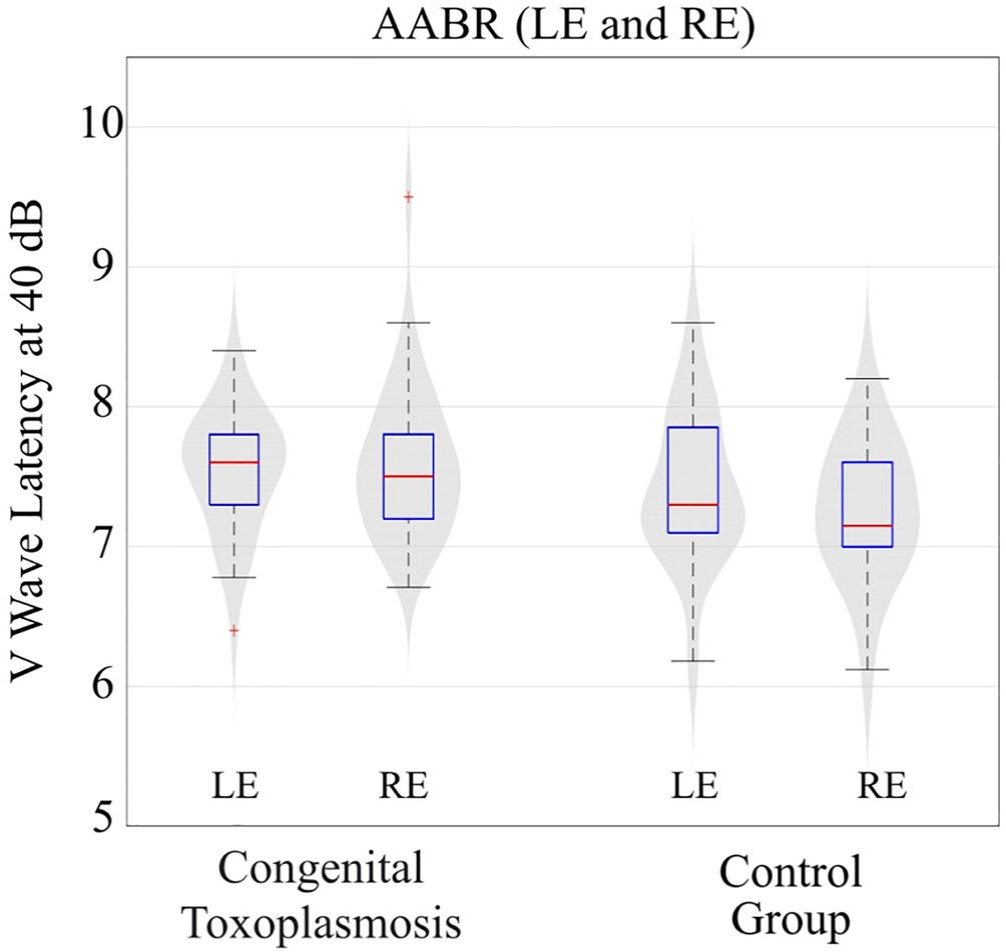
Analysis of wave V (ms) latency found in the AABR at 40 dB in the two groups studied. The points in red identified in our boxplot figure are outliers, that is, two extreme values were observed in our analysis of wave V of the AABR (*LE* left ear; *RE* right ear).

However, the AABR measurements showed differences in wave V latencies at 40 dBHL (Fig. 1). The latency of wave V in the right ear showed a significant difference between groups with a tendency for greater latencies in the CT group compared to the control group. The Mann–Whitney test showed that the group with CT infection has an effect on wave V latencies in AABR (U = 259.5; p < 0.05) in the right ear, but it does not show the same in the left ear (U = 315.0; p > 0.05).

### Functional near-infrared spectroscopy (fNIRS)

**Figure 2:**
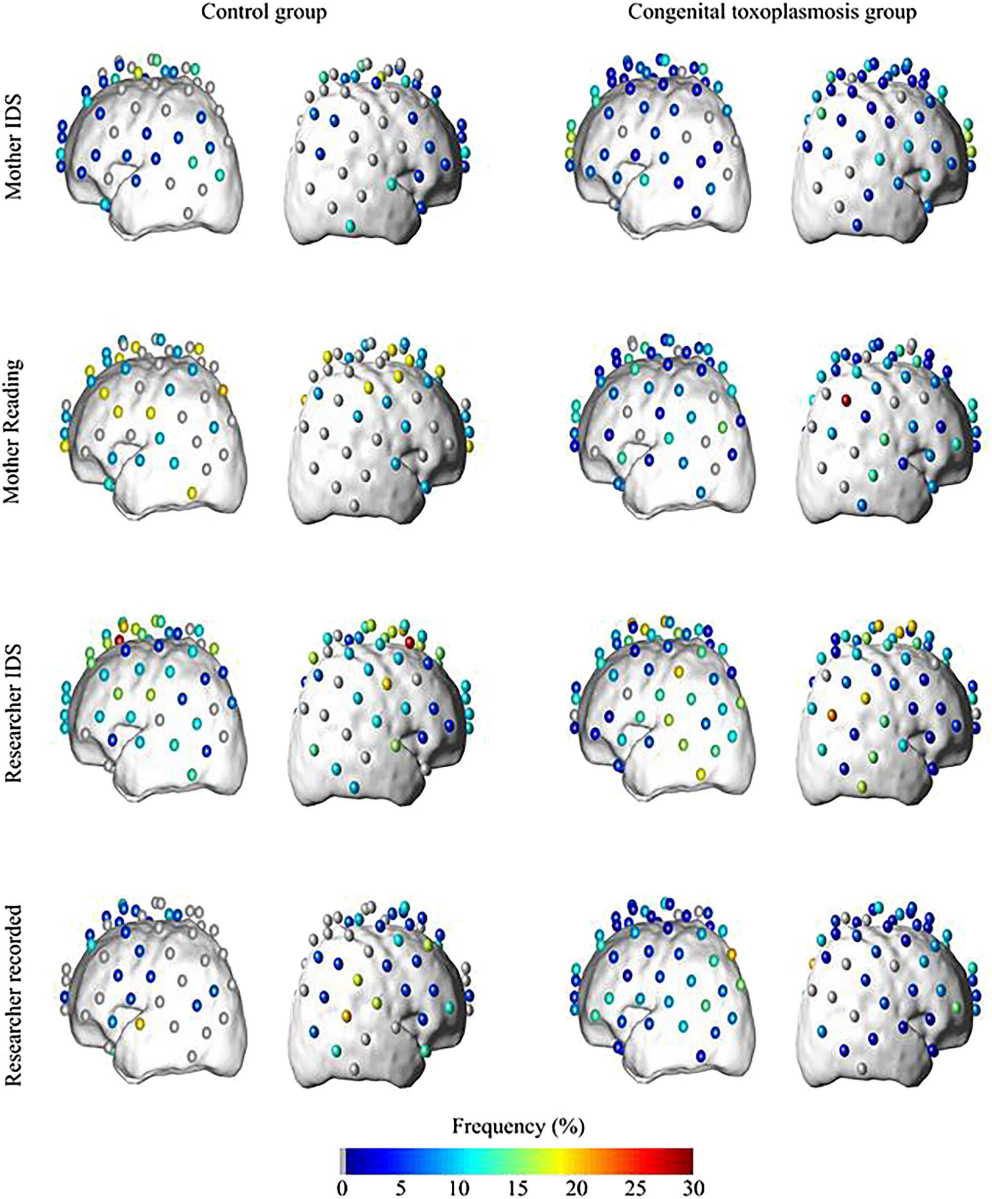
Frequency of activated channels in control group for: mother infant directed speech (IDS) n = 16; mother reading n = 10; researcher infant directed speech n = 17; researcher recorded n = 16; and in Congenital toxoplasmosis group for: mother infant directed speech n = 24; mother reading n = 23; researcher infant directed speech n = 27; researcher recorded n = 30.

For the control group (23 infants), the proportion of infants that had at least one activated channel was 90% for mother reading, 78.9% for researcher infant directed speech (IDS), 58.8% for mother IDS, and 70.6% for researcher recorded stimuli. For the Congenital toxoplasmosis group, we observed that 79.2%, 74.2%, 85.7%, and 68.8% of all infants (38 subjects) had at least one activated channel for mother reading, researcher IDS, mother IDS, and researcher recorder stimuli, respectively. In both groups, we found hemodynamic responses to speech stimuli on temporal, parietal and frontal regions in both hemispheres, and a small frequency of activation and deactivation in each channel. Figure 2 shows the pattern of activation (frequency maps) in both groups for all stimuli. In the control group, the channels with the highest frequency of activation were located in the right posterior frontal, right posterior temporal, left posterior parietal and right anterior parietal brain regions for the tasks researcher IDS, researcher recorded, mother reading and mother IDS, respectively. Interestingly, even the highest frequency is surprisingly low (29%). The low frequencies combined with the fact that, on average, 75% of volunteers included had at least one activated channel, corroborates with the less specific brain area pattern seen in Fig. 2. In fact, even though each stimulus evoked characteristic hemodynamic responses in the brain cortex of most of the volunteers, the activated channels varied within the group. We observed a similar behavior for the Congenital toxoplasmosis group. Although most (77%) of the volunteers presented at least one activated channel, the range of highest frequencies varied from 18% (task mother IDS) to 32% (task mother reading).

**Figure 3:**
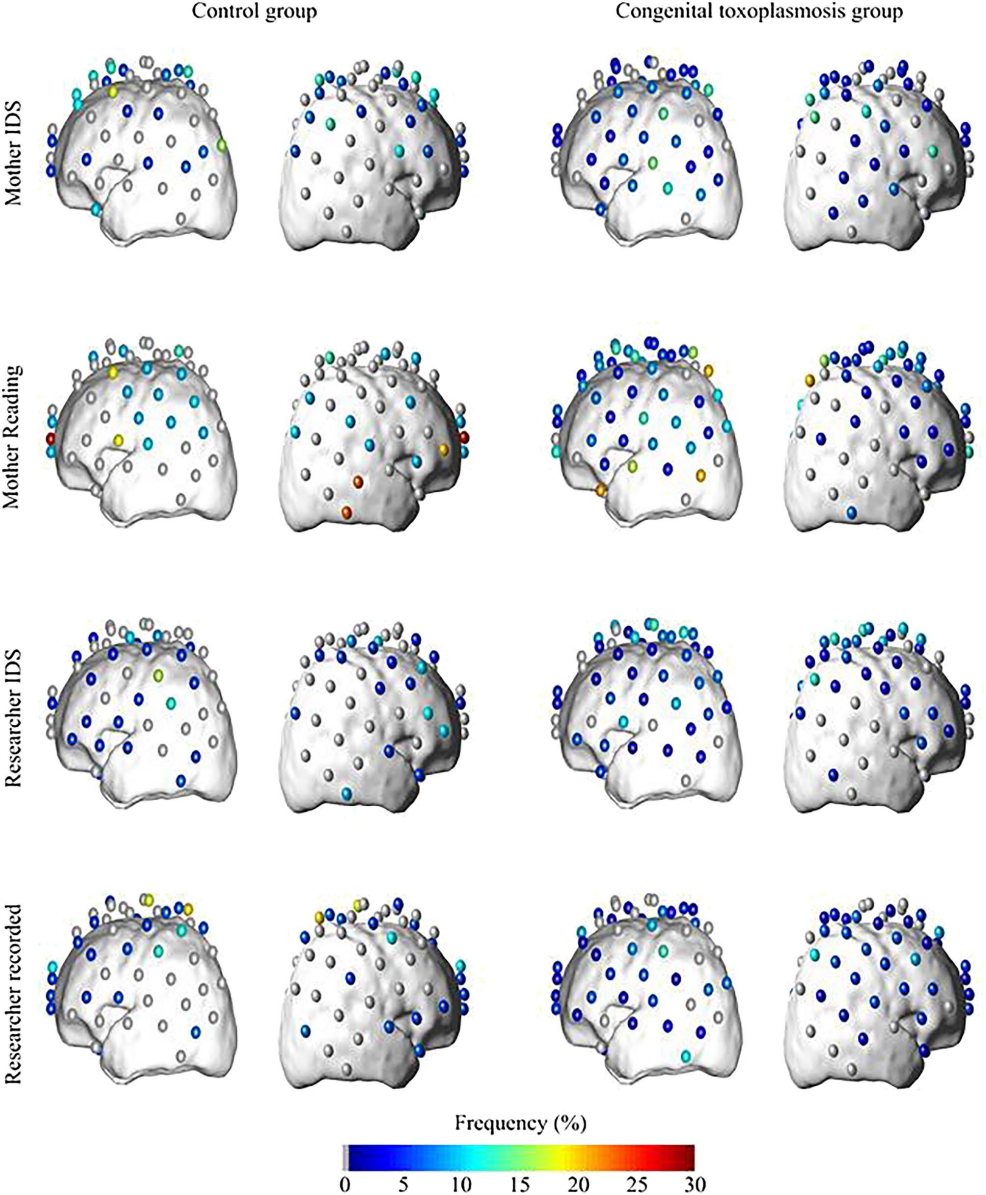
Frequency of deactivated channels in control group for: mother infant directed speech (IDS) n = 16; mother reading n = 10; researcher infant directed speech n = 17; researcher recorded n = 16; and in Congenital toxoplasmosis group for: mother infant directed speech n = 24; mother reading n = 23; researcher infant directed speech n = 27; researcher recorded n = 30.

When analyzing the deactivation pattern for the four stimulus conditions in both groups (Fig. 3), in the control group, the channels with the highest frequency of deactivation were located in the left prefrontal, upper left parietal, left posterior frontal and left parietal brain regions for the tasks mother reading, researcher recorded, mother IDS, and researcher IDS, respectively. In fact, even though each stimulus evoked characteristic hemodynamic responses in the brain cortex of most of the volunteers, the deactivated channels varied among them, suggesting that each child responded in his or her own way to the external stimuli. The same behavior was observed for the Congenital toxoplasmosis group. Although 62% of the volunteers presented at least one deactivated channel, the range of highest frequencies of deactivation varied from 14% (tasks researcher recorded and researcher IDS) to 23% (task mother reading).

Given the high variability in the location of the hemodynamic responses within each group for every stimulus, we decided to avoid comparisons between groups. Instead, we focused on verifying if there were changes in the number of recruited brain regions (or NIRS channels) across stimuli within the groups. We used the nonparametric Wilcoxon Rank Sum test for assessing this difference considering only the number of activated and deactivated channels (separately). We found no significant differences for any task within each group in any situation. Therefore, it appears that although there is a high variability in the location of the hemodynamic responses within group for each task, the distribution of the number of activated or deactivated channels does not change across stimuli within the same group.

## Discussion

Most newborns with Congenital toxoplasmosis are asymptomatic^9^, and early treatment seems effective to minimize neurological sequelae, allowing a better brain development and growth^7–9^. Some neurological and hearing changes were found^5–7^. We observed 31.6% of children affected with calcifications, hydrocephalus or any finding on neuroimaging even after early treatment. But even with diagnosed neurological lesions, we found no significant differences in overall auditory screening outcomes (pass/fail results) except for a more prolonged wave V latency in the CT group compared to control group. The AABR allows the identification of retrocochlear alterations and to assess the maturational status of the central auditory nervous system in infants. Its sensitivity for detecting such problems is great, since it does not depend on the patient's information. Hall et al. found that AABR has 100% sensitivity and specificity of 99.7%^23^.

The focus of this study was not to identify or analyze hearing loss in children with CT, but to evaluate how the brain responds to auditory stimuli having a white matter disorder. All children had wave V present at 40 dBHL in AABR (which means normal hearing sensitivity). However, we found an increase in wave V latency in the group with congenital toxoplasmosis when compared to the control group. Wave V latency reflects synchrony and time of response to auditory stimulus at brainstem level, more specifically inferior colliculus neuronal activity. The maturation of the auditory system directly influences the AABR responses of infants, and its response changes along the months of life^24,25^. The progressive myelination of structures of central nervous system, resulting from the maturational progress of the central auditory system, leads to the improvement of the synchrony of neural activity and greater functionality of synapses, resulting in decrease in wave V latency^26^. This result shows a possible link between the increase in AABR latency and infection with *T.gondii*, further emphasizing a potential change in maturation of the central auditory nervous system.

According to Sleifer et al.^13^, in individuals with normal peripheral hearing the responses of both ears in brainstem audiometry are similar, since the anatomical structures involved are in the brainstem, that is, structures used by both ears when sound stimulation occurs. Therefore, we standardized to use the data from the right ear, as we had all records of absolute wave V latencies in the AABR and these results were within normal limits. These auditory electrophysiological findings suggest a possible different pattern of response to any auditory stimuli in brains of CT group. However, we could not observe differences in the auditory response between CT and health individuals within the early months of life.

Considering that the stimuli processing might be changed by the neuronal specialization, these findings indicate the need for regular monitoring of the hearing skills of children with Congenital toxoplasmosis or related outcomes. Monitoring the development of hearing and language in these children may be fundamental to find a prognostic value and early identification of changes in AABR^17^.

Here, we tested how the brain of healthy and Congenital toxoplasmosis infants perceived speech in four different auditory-speech stimuli. We used mother IDS, researcher IDS, mother reading and researcher recorded stimuli to record the cortical activation pattern. Several fNIRS studies have used speech stimuli to understand brain activation in young children^27–34^. Newborn brain seems to be able to make relations between linguistic units and their sequential position, and integrate this information into a coherent representation of language structure^27^. They seem to discriminate between a familiar and an unfamiliar language^28^, and between a normal speech and a highly compressed speech^29^. The brain response to a forward speech is different to a backward speech for a maternal language^30,31^ and has a syllabic sonority-related preference as seen in adults^32^. Information from multisyllabic sequences can be encoded^33^ and recognized^34^. Using non-speech stimuli, there is no evidence of hemispheric asymmetries at birth^35^, but it is possible to find clear responses to different prosodic group patterns based on variation of duration, pitch and intensity of the stimuli^36^, and to the right-lateralized responses to slow modulations sounds in the first months of life^37^.

We observed a less specific cortical activation pattern in both groups. Most of the infants had less than 3 channels (out of 70 channels) activated during the stimuli, and most of the activated channels differed across the infants resulting in a low frequency of activation per channel. Based on previous findings, we expected that infants born with full auditory processing abilities would activate specific cortical areas^22,34,36,38^. Indeed, a widespread pattern of activation to auditory input was found in 3-month-old infants^38,39^, suggesting a non-specific brain response at this age. In our findings, even a child with a congenital inflammatory disorder has the same brain activation as a typical child, which may be related to the high levels of brain plasticity at this life stage. Watanabe et al. studied audio-visual stimuli in 3-month-old infants and found that auditory input induced specific changes in each cortical region. This result shows that, at this age, children may perceive multisensory inputs by using a global network of functional cortical areas^40^. Watanabe et al. found that visual stimuli activates several regions of the cortex in 2-month-old infants and induces functionally differentiated regions in 3-month-old children’s cortex, suggesting a general-to-specific response through the cortical development at this period related to the stimuli^41^. Kotilahti et al. found bilateral brain activation and no significant responses to speech or music stimuli in newborns^42^.

A recent study using diffusion tensor imaging and resting state connectivity analysis in children with profound sensorineural hearing loss, and consequently no hearing experiences, identified widespread white matter changes that might have modified auditory and language pathways^43^. From the studies of sensorineural hearing loss, there is a suggestion that the less specific brain area pattern was the result of axonal loss, lack of myelination and less fibers projections in children related with auditory deprivation^43^. We need more studies to further understand the potential damage due to brain lesions identified in CT.

During the maturation process, less pathways are activated by a specific stimulus because most of them are tuned to other functions, so the area activated by this stimulus becomes smaller^57^. As suggested by previous studies using NIRS and different auditory stimuli to understand the activation pattern in infants, there is a curve of cortical specialization to stimuli as speech and social sounds over the development of the child^22,38,44,45^. Later, there is a localized and selective brain response for social sounds stimuli from 9 to 24 months of age but not from 0 to 2 months of age^44^. When comparing children with 3 and 6 months of age, there is a widespread pattern of brain activation to communicative human vocalizations at 3 months, while infants showed more focal responses to this stimulus at 6 months^45^. Fava et al. showed that 3-to-6-month-old infants have a greater bilateral cortical activity in response to non-native speech compared to native speech, both infant-directed and audiovisual stimuli. In an 11-to-14-month-old infant, the processing was left lateralized and with a strong response to native compared with non-native speech^38^ showed bilateral cortical activity that was greater in overall response. We also found a heterogeneous brain deactivation in both groups. Deactivation may be caused by the ‘‘stealing’’ of activation by a neighboring area, that is, the elevation of blood flow at the activated location causes reductions in blood supply in nearby areas sharing the same blood vasculature^46–49^.

The *Toxoplasma gondii* has an affinity for the central nervous system (CNS)^5^. During pregnancy, the parasite overcomes the restrictiveness of the blood–brain barrier and establishes an infection in the CNS. We hypothesized that both the inflammatory processes and the cortex calcifications in children with Congenital toxoplasmosis would make brain response to hearing more unspecific, recruiting more cortical areas for sensory processing. However, the fact that infants had a broad response in all cases, with different activated regions and no standard pattern within the group, makes comparison between the CT and control groups of secondary importance. The heterogeneity in response might result from the effectiveness of the early treatment, or simply being a limitation of the measurement made at a period in which the brain is very plastic and is still under natural development.

The reduction of morbidity and mortality of CT in children of treated mothers and the profound impact of infection-induced maternal immune activation during pregnancy on developing neural circuits^50^ have encouraged governments, health professionals and researchers to invest in early identification of infection of *T gondii* and in prophylactic health actions^2–4,6–9,51^. When the treatment of a pregnant woman started within 3 weeks after maternal seroconversion, the odds of mother-to-child-transmission were 52% lower when compared to delayed treatment^52^. Although it is possible to find a reduction of intracranial calcifications related to treatment^53^, we observed 31.6% of affected children with calcifications, hydrocephaly and others findings in neuroimaging. These clinical findings may compromise these children’s neurodevelopment. Understanding how the brain is modified by the infection and its treatment effect might help to clarify what is still necessary to improve the outcomes of the affected children. There is a clear need to follow up these children to better understand the potential compromise of important outcomes as language and auditory measures.

When considering the development of the auditory-linguistic system of an infant with CT, studies have revealed a higher risk to have an altered brainstem auditory potential^17^, higher latency and differences in the auditory nervous system to encoding of speech^16^ and language delays^10^, when compared to typical children. Although sensorineural hearing loss has a rare occurrence in treated children^9^, it is recommended that these children have a follow-up audiometric evaluation at least until 24 to 30 months of age^6^. The development of speech and language skills occurs concurrent with the maturation of the auditory function, since the period of reception of auditory language indicators is a prerequisite for the later application of language^54^. There is a 1.59 higher risk to develop a learning disorder after infection with Toxoplasma that is still not well understood in neuropathology of brain lesion caused by *Toxoplasma gondii*^55^*.*

This study observed brain activation in response to four auditory stimuli in infants. Further studies are necessary to understand the interaction between the congenital infection, brain injury and children outcomes. There are some limitations to our study derived from working with infants, because we had to adapt the stimuli and the child’s general condition during the exam, since most stimuli were natural and spontaneous, and infants had different conditions (alert, sleepy, sleeping, breastfeeding). These differences certainly interfered in the patterns of cortical activation. Although we describe these factors as limiting ones, they also may be seen as a strength point since they reflect natural day-to-day conditions in the listening environments related to infants’ behaviors. Finally, we had a small sample that may not have a strong representation of infants with Congenital toxoplasmosis. Larger studies will be able to minimize interference from children's conditions.

Congenital toxoplasmosis has been investigated since the 1950s, but it still comprises an understudied field. One of our constraints might be due to the maturational course of the central auditory nervous system. For CT, we studied a group prospectively followed-up, which received early diagnostic and treatment for the infection, minimizing possible damages caused from the congenital infection. Nonetheless, these babies often have microcalcifications that could lead to a different pattern in sound and speech perception, such as less specialized responses, or even less strength (Hb concentration levels) in activated areas during auditory stimulation. For further studies, it is crucial to perform longitudinal assessments with CT to monitor neurodevelopment with focus on auditory and language outcomes.

## Materials and methods

### Participants

Sixty-one full-term infants and their mothers participated in the present study (34 boys and 27 girls; average age = 57 days; range: 17–94 days, SD: 22 days). Five babies were excluded because they showed signs of discomfort during the experiment.

Thirty-eight infants with Congenital toxoplasmosis (n = 38; average age = 58 days, range 18–94; 16 females) were followed up in the Program for Congenital Toxoplasmosis Control. This is a public health program in Brazil which performs the postnatal diagnosis of Congenital toxoplasmosis and follow-up children up to 12 years of age^51^. Children are diagnosed and treated according to clinical protocols and then annually followed-up by a multidisciplinary team. All babies enrolled in this study had IgM. anti- *T gondii* positive during the neonatal screening test in dry blood and had positive serology (IgG and IgA/IgM anti- *T gondii*). Children born with gestational age bellow 36 weeks at birth, children with neurodevelopment delay, hearing disabilities, a clinical or imaging sign of neurologic disorder not related to CT, syndromes or other congenital infections were excluded from this study. All cases of Congenital toxoplasmosis were early diagnosed and treated.

Twenty-three healthy infants (n = 23; average age = 56 days, range 17–90; 11 females) composed the control group. They were both children who had a progressive decline in IgG and were excluded from the program due to the negative diagnostic confirmation of CT, and children recruited from the neonatal hearing screening program of the state. No hearing disabilities, neurodevelopment delay, prenatal or perinatal complications were reported from the parents for any of the participants in this group.

Table 2 shows the demographic characteristics for the groups. Infants in the two groups did not differ on gestational age, birth weight, postnatal age, and head circumference.

**Table 2:** Demographic characteristics of the children enrolled in this study.

	CT (n = 38)		Control (n = 23)		p-value^a^
	Mean	SD	Mean	SD	
**At birth**					
Gestational age (weeks)	38.5	1.03	38.9	1.04	0.178
Birth weight (g)	2992.10*	931	3255.50**	966	0.181
Gender (F/M)	16/22		11/12		0.669
**At NIRS**					
Postnatal age (days)	58	22	56	21	0.707
Head circumference (cm)	37.53	2.07	38.08	2	0.319

*CT* congenital toxoplasmosis; *SD* standard deviation.

*10 subjects without information.

**4 subjects without information.

^a^Student t test (p < 0.05).

Neuroimaging tests from the CT group showed normal tests for 25 (65.8%) infants and calcifications in 10 (26.3%) infants. Less frequent findings were peri-intraventricular hemorrhage (2.6%), hydrocephalus (2.6%), demyelination signs (2.6%), cerebral atrophy (2.6%) and ventricular dilation (2.6%).

All methods were carried out in accordance with relevant guidelines and regulations and this study was approved by the ethical committee of the Federal University of Minas Gerais (nº 2.277.701, CAAE: 45493415.6.0000.5149). Signed informed consent was obtained from the parents.

### Procedures

#### Hearing assessment

Hearing assessments were carried out in an acoustically treated room, in the presence of the parents and/or person responsible for the infant. Transiently Evoked Otoacoustic Emissions (TEOAE) and Automated Auditory Brainstem Responses (AABR) comprised the audiologic test battery. Data were collected with Elios handheld equipment from Echodia. The TEOAE measurements were performed with click stimuli at sound pressure levels of 80 dBSPL, in a window of 20 ms.

AABR was recorded with 0.1 ms duration alternated click stimulus at a presentation rate of 17.1 stimuli per second. Artifacts control was set during the recordings allowing a maximum of 10%. Results were considered normal if wave V was present at 40 dBHL and latency within prescribed limits according to normality criteria from the device.

All children included in the study had normal results in the hearing assessment. Some children (n = 3) from the control group did not perform AABR. Nonetheless all of them had normal cochlear responses in the TEOAE.

For statistical analysis, the SPSS 15.0 program was used (SPSS, Inc., Chicago, IL, United States of America). Descriptive analysis of wave V latency values are provided (mean and standard deviation). As latency values were asymmetrically distributed (Shapiro–Wilk), comparison between control and CT groups was performed with Mann–Whitney test and p-value < 0.05 for significance.

#### Experimental protocol

During data acquisition, all infants stayed on their mother’s lap throughout the whole experiment. Some of them were breastfeeding or in a state of light sleep. The experimental protocol accounted for four different auditory-speech stimuli. Each stimulus was recorded on a separated run. All stimuli were block-designed with an average of six blocks (range 4 to 9) of auditory-speech stimuli (details in the following section). Each block had one period of stimulation that varied from 10 to 13 s followed by a resting period of a minimum of 10 s. In some cases, in which the babies were not quiet after the resting period, we extended the period of resting until the babies remained quiet for at least 10 s. The extra time was important to guarantee a clean baseline period prior to each trial.

We selected four different auditory-speech stimuli: mother infant direct speech (mother IDS), researcher infant directed speech (researcher IDS), mother reading and researcher recorded. In the mother IDS stimulus, each mother talked to her baby for 10 s as she usually does at home. In the researcher IDS, a trained female researcher talked to the babies for 10 s with infant directed speech. The voice of the researcher was set at approximately 60 dBSPL, and the researcher talked from 40 cm from the baby. In the mother reading stimuli, each mother read a small excerpt from the fairy tale “Little red riding hood’’ for an average time of 12 s, with adult directed speech. In the last stimulus, researcher recorded, we presented a 12-s recording performed by a female researcher who read a fragment from the “Little red riding hood’’ aloud, also with an adult directed speech. We presented the recording through a speaker with 65 dBSPL that was located about 50 cm away from the infant. A sound pressure level meter app was used to estimate intensity. All verbal stimuli were presented in Portuguese.

#### Functional near-infrared spectroscopy (fNIRS) data acquisition

We performed all measurements with a commercial continuous-wave (CW) near-infrared spectroscopy system (NIRScout Tandem 1616, NIRx Medical Technologies, Glen Head). We built our optical probe with 30 sources (each source contains 2 LEDs centered at 760 and 850 nm) and 28 detectors, allowing 84 source-detector separations that ranged from 1.5 to 2.5 cm. However, due to bad functioning of some sources from optical system on final experiments, we opted to exclude seven channels from each brain hemisphere from the occipital lobe from all subjects. This exclusion led us to an optical probe with 70 channels, covering the frontal, temporal, and parietal lobes. We performed all measurements at an acquisition frequency of 4 Hz. We held the optodes onto the volunteer’s head with a 10–20 standard head cap. To adjust the head cap in each subject’s head, we used a measuring tape and followed a standard approach that considers the subject’s head circumferences, nasion-inion, and ear-to-ear distances as a guide to position the optical probe^56,57^.

### Data processing and analysis

**Figure 4:**
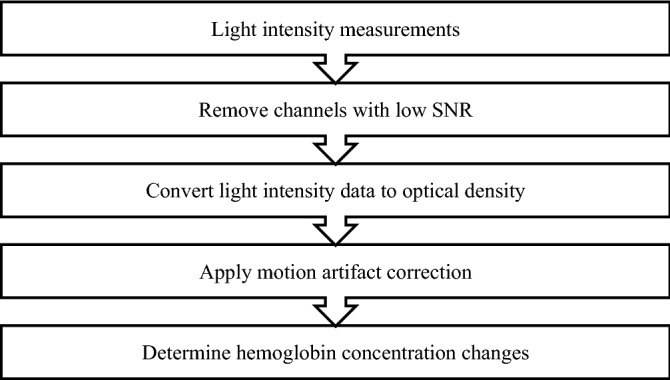
Adapted from the study by Novi et al., Workflow of the preprocessing steps of fNIRS data^63^.

We processed and analyzed all fNIRS data with homemade MatLab (Mathworks, Inc., Natick, MA, USA) scripts based on HomER2 codes^58^. First, we discarded channels with low signal-to-noise ratio (SNR). In this work, we employed an SNR greater than or equal to 4, *i.e.* a given channel was considered as of appropriate quality if its mean intensity was at least 4 times greater than its standard deviation for both wavelengths. This choice of SNR was based on visual inspections of the raw time-series and on our previous experiences on adult and infant data acquired with the same fNIRS systems^59–61^. Since we wanted to investigate the evoked hemodynamic responses in different brain regions, we opted to exclude volunteers that did not have at least 40 channels with good SNR. For the control group, this last criterion led to the exclusion of 5 out of 15, 7 out of 23, 7 out of 23, and 6 out of 23 subjects for the mother reading, researcher recorded, mother IDS, and researcher IDS, respectively. For the case group, the additional exclusion criterion led to the exclusion of 5 out of 28, 8 out of 38, 14 out of 38, and 11 out of 38 volunteers for the mother reading, researcher recorded, mother IDS, and researcher IDS, respectively (Fig. 4).

Next, we converted light intensity of each remaining channel to optical density (OD), and then we removed motion artifacts in the OD time-series with a hybrid method that combines spline interpolation and wavelet decomposition^62,63^. After motion artifact correction, we computed hemoglobin concentration changes using the modified Beer-Lambert law with a differential pathlength factor of 6 for both wavelengths^64,65^. Next, we band-pass filtered hemoglobin concentration changes between 0.001 and 0.8 Hz to remove slow drifts and high-frequency noise. Finally, for each channel of each volunteer, we verified if the channel had a characteristic hemodynamic response function (hrf) with an adaptive general linear model (GLM)^66^. The expected hrf should present an increase in oxy-(HbO) and decrease in deoxy-hemoglobin (HbR) synchronized with the period of stimulation. Channels with significant (p < 0.05) increase for HbO and significant decrease for HbR were classified as activated. As a complementary analysis, we also inspected which channels presented an opposite behavior of the typical hrf. In other words, we repeated the same adaptive GLM analysis, but channels with a decrease in HbO and an increase in HbR were defined as deactivated.

To summarize the results across subjects, we created frequency maps of activation and deactivation. Each frequency map of activation and deactivation was created independently for each task and group. To compute the frequency maps of activation, we first create a vector (**v**) that counts the number of times that each channel was assigned as activated for a given task across all volunteers within a group. Next, we divide **v** by the number of volunteers of the group, resulting in a vector of frequency (or frequency map). The same procedure is done for creating frequency maps of deactivation. We decided to use frequency maps of activation and deactivation because frequency maps are more meaningful than averages across volunteers when the variability within group is high^59–61^. Finally, we compared differences among auditory stimuli within each group via the nonparametric Wilcoxon Rank Sum test considering only the distribution of the number of activated and deactivated channels per group and per task.
